# M2 macrophage polarization modulates epithelial-mesenchymal transition in cisplatin-induced tubulointerstitial fibrosis

**DOI:** 10.7603/s40681-016-0005-5

**Published:** 2016-02-22

**Authors:** Chia-Cherng Yu, Chiang-Ting Chien, Tzu-Ching Chang

**Affiliations:** 1 Department of Medical Research, National Taiwan University Hospital, 100 Taipei, Taiwan; 2 National Taiwan University College of Medicine, 100 Taipei, Taiwan; 3 Department of Life Science, National Taiwan Normal University, 106 Taipei, Taiwan; 4 Metabolomic Research Center, China Medical University Hospital, 404 Taichung, Taiwan; 5 Graduate Institute of Clinical Medical Science, China Medical University, 404 Taichung, Taiwan

**Keywords:** M1/M2 macrophage polarization, Epithelial-Mesenchymal Transition, Cisplatin, Renal tubulointerstitial fibrosis

## Abstract

Cisplatin-induced nephrotoxicity leaded to apoptosis of tubular epithelial cells (ECs) and tubulointerstitial fibrosis through ROS stress and inflammatory cytokines. Tubulointerstitial fibrosis caused by cisplatin might be *via* activation of resident fibroblasts and epithelial-mesenchymal transition (EMT) of tubular ECs. Inflammatory niche was crucial for progression of fibroblast activation or EMT. It had been reported that M1/M2 macrophage polarization regulated pro-inflammation or pro-resolving phase in damage repairing. However, the role of macrophage polarization on cisplatin-induced EMT of tubular ECs had not been well elucidated. In this study, we used co-cultured cell model and condition medium to examine the interaction between tubular ECs, fibroblasts and M1/M2 macrophages. Our data showed that cisplatin alone induced incomplete EMT of tubular ECs, whereas fibroblasts co-cultured with cisplatin-treated ECs could lead to fibroblast activation by detection of α-SMA and collagen-1. Moreover, decrease of iNOS and increase of argenase-1 and CD206 expression indicated that macrophages co-cultured with cisplatin-treated ECs would turn to M2 phenotype. Finally, we found that condition medium of M2 macrophages could promote complete EMT of cisplatin-treated ECs. Taken together, cisplatin created an inflammatory niche via tubular ECs to activate fibroblasts and stimulated M2 macrophage polarization. M2 macrophages could turn back to promote EMT of cisplatin-treated ECs. These results revealed the cooperative roles of tubular ECs, fibroblast and M2 macrophages to facilitate the progression of renal fibroblasis.

## 1. Introduction

Renal fibrosis, including glomerulosclerosis or tubulointerstitial fibrosis, may be caused by ischemia-reperfusion (IR) injury or some neurotoxicity such as cisplatin [1-4]. Cisplatin (cisdiamminedichloroplatinum (II)), an effective anti-cancer drug, have severe side effects of nephropathy [5-9]. The mechanisms of cisplatin in cancer therapy rely on its crosslinking with and disrupting DNA, inducing mitochondria damage, oxidative stress, cell cycle arrest in G2 phase and cell apoptosis. Furthermore, cisplatin-induced nephrotoxicity is through acute cytotoxic effects on tubular epithelial cells, resulting in loss of tubular epithelial cells by apoptosis, necrosis and loss of cell adhesion followed by inflammatory cell infiltration and fibroproliferative changes [7 and 9].

The pro-inflammatory cytokines secreted by immune or damaged tubular epithelial cells lead resident fibroblasts turning to active myofibroblast [10-11]. Active myofibroblasts express α-SMA, secrete more collagen I and make other aberrant matrix synthesis and deposition. Although the most of active myofibroblasts come from resident interstitial fibroblasts, some reports have showed at least 30% of total myofibroblast population origin from tubular epithelium [12-16]. The proposed mechanism involved in these phenomena is epithelial-mesenchymal transition (EMT). Loss of epithelial cell adhesive properties and gain of fibroblast-like characteristics are the typical process of EMT. The major stimulations of EMT are some kinds of cytokines such as TGF-β or FGF. Once EMT is induced in renal tubular epithelial cells, it declines E-cadherin expression, induces the expression of fibroblast markers such as vimentin and fibronectin, destroy the tubular basal membrane and transmigrate into renal interstitial spaces [12-16]. Some studies have reported that EMT of tubular epithelium may be involved in cisplatin-induced renal fibrosis [11, 13, 17, 18]. Cisplatin-elicited ROS stress in damaged epithelial cells and pro-inflammatory cytokines secreted by surrounding immune cells are the possible causes of EMT. Yamamoto et al have demonstrated ciaplatin-induced EMT of tubular epithelial cells and indicated that PGE_2_ inhibit epithelial apoptosis and EMT to improve epithelial regeneration [17]. Moreover, Benedetti et al report that NFκB facilitate cisplatin/TNFα synerge-induced epithelial apoptosis by suppression of EMT [18].

Macrophages are the major immune cells in inflammatory niche during inflammation and recovery phase [19-21]. However, the roles of macrophages have not been well elucidated in cisplatin-induced EMT. Some researches propose that there are two subtypes of macrophages, called classical activation type I and alternative activation type II macrophages [22-26]. Type I is considered as pro-inflammatory macrophages since they secrete pro-inflammatory cytokines such as TNF-α and INF-γ. Type II is considered as anti-inflammatory or repaired macrophage since they secrete anti-inflammatory cytokines such as IL-10 and TGF-β [25, 26]. Depletion of macrophages by lipososme clodronate significantly attenuated rat renal fibrosis caused by ischemia-reperfusion injury (IRI) [27]. Macrophages shifting from pro-inflammatory M1 to pro-resolving M2 have been demonstrated to help kidney repair following IRI [28]. Moreover, Kim et al have recently demonstrated that M2 macrophages were more important than M1 in the development of fibrosis following IRI [29]. In this study, we use co-culture cell models in vitro to examine the role of M1/M2 macrophage polarization on cisplatininduced epithelial EMT.

## 2. Methods and Materials

### 2.1. Cell culture

Raw 264.7 cells (from ATCC) and mouse tubular epithelial cells (from ScienCell) were routinely cultured in Dulbecco’s modified Eagle’s medium (DMEM) and supplemented with 10% fetal bovine serum (Hyclone), 100 U/ml penicillin and 100 μ;g/ml streptomycin at 37°C in a humidified 5% CO_2_ atmosphere. Cisplatin were purchased from Sigma-Aldrich.

### 2.2. Western blot analysis

Western blotting was performed as previously described [30]. Rabbit monoclonal antibodies against E-cadherin and Snail2 were from Cell Signaling. Rabbit monoclonal antibodies against vimentin and rabbit polyclonal antibody against fibronectin purchased were from abcam.

### 2.3. Quantitative real-time PCR (qPCR) analysis

Total RNA was isolated using TRIzol reagent (Invitrogen) according to the manufacturer’s instructions. RNA concentration was quantified with a NanoDrop ND-1000 Spectrophotometer (Nanodrop Technologies) and RNA quality was checked with the ratio of 260/280. Quantification of mRNA expression for candidate genes was performed by qPCR using ABI One-step Detection System Instrument (Applied Biosystems). Total RNA was reverse-transcribed by using high capacity cDNA reverse transcription kit (Invitrogen). qPCR reactions were performed with the power SYBR Green PCR Master mix (Roche) in a MicroAmp optical 96-well reaction plate according to the manufacturer’s instructions. Relative gene expression levels were normalized to GAPDH expression. The primer sequences of each genes for qPCR were as follows. Mouse *α-SMA*: (F) 5’CAGGGAGTAATGGTTGGAAT3’, (R) 5’TCTCAAACATAATCTGGGTCA3’; mouse *Collagen-1*: (F) 5’CCTGGTAAAGATGGTGCC3’, (R) 5CACCAGGTTCACCTTTCGCACC- 3’; mouse *iNOS*: (F) 5’CGAAACGCTTCACTTCCAA3’ (R) 5’ TGAGCCTATATTGCTGTGGCT 3’; mouse arginase-1: (F) 5’AACACGGCAGTGGCTTTAACC3’, (R) 5’GGTTTTCATGTGGCGCATTC3’; mouse CD206: (F) 5’CAGGTGTGGGCTCAGGTAGT 3’, (R) 5’TGTGGTGAGCTGAAAGGTGA 3’.

### 2.4. *In vitro* induction of macrophages M1 and M2 from peripheral blood monocytes (PBMCs)

Fresh peripheral bloods were collected in a defibrinated state from mice, diluted with PBS and then Ficoll-Paque added. Centrifuged at 400 g for 30 min at 20°C. Draw off upper plasma layer and collected middle monocyte layer by sterile pipettes. These isolated monocytes were then cultured in Macrophage generation DXF (from PromoCell) to develop into general macrophages. Then these macrophages were treated with INF-γ and IL-4 to induce into M1 and M2 macrophages respectively.

### 2.5. Statistical analysis

Differences between groups were analyzed by Student *t* test. A *P* value of less than 0.05 was considered statistically significant.

## Results

### 3.1. Tubular epithelial cells treated cisplatin alone underwent incomplete EMT

**Figure 1:**
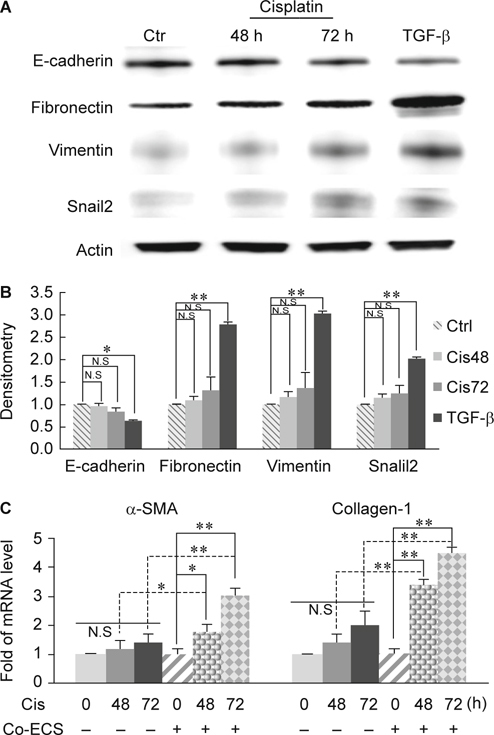

To examine the effects of cisplatin on EMT, we treated tubular epithelial cells (ECs), PK, without or with 20 uM cisplatin for 48 and 72 hours (h), respectively. Markers of EMT included E-cadherin, fibronectin, vimentin and snail2 were detected with western blotting. Cells treated with cisplatin for 48 h displayed no significant changes in E-cadherin, fibronectin, vimentin and snail2 (Fig. 1A-B). Moreover, 72 h treatment of cisplatin also showed no significant changes in E-cadherin and snail2. Although cisplstin induced slight increase of fibronectin and vimentin at 72 h treatments, the statistics of densitometry analysis demonstrated no significant changes (Fig. 1A-B). These results indicated that cisplatin alone induced incomplete EMT of tubular epithelial cells.

### 3.2. Fibroblasts co-cultured with cisplatin-treated ECs turned to myofibroblast

In addition to EMT of tubular ECs, the other major cause of tubulointerstitial fibrosis is the activation of resident fibroblasts. To understand whether cisplatin had direct effects on fibroblast activation, we treated fibroblasts with cisplatin alone or co-cultured with cispltin-treated ECs to mimic the inflammatory niche. We then detected mRNA expression levels of two major markers of fibroblast activation, α-smooth muscle actin (α-SMA) and collagen- 1. Fibroblasts treated with ciaplatin alone, no matter how long the fibroblasts were incubated (48 or 72 h), both the mRNA levels of α-SMA and collagen-1 had no significant changes while compared with control (Fig. 1C). However, while co-cultured with cisplatin-treated ECs, fibroblasts turned to activate and both the mRNA levels of α-SMA and collagen-1fibroblasts had been significantly increased (Fig. 1C). It implied that an inflammatory niche is more crucial than a toxic reagent alone on activation of fibroblasts. Taken together, cisplatin alone could not lead to complete EMT of tubular ECs but it supported an inflammatory niche through ECs to activate fibroblasts.

### 3.3. Co-culture with cisplatin-treated ECs led to M2 macrophage polarization

**Figure 2:**
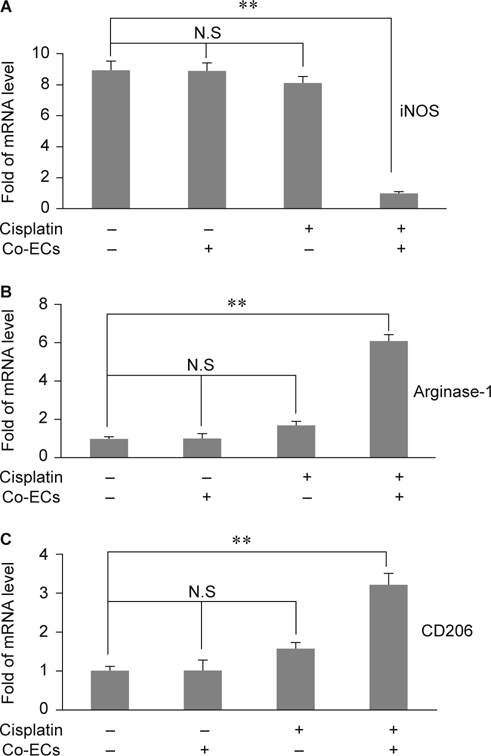

Macrophages played an important role on acute and chronic inflammation. Recently, macrophage polarization had been reported to contribute the fibrosis progression. However, the role of macrophage polarization in cisplatin-induced fibrosis is not clear. According to the above data, we wonder if the inflammatory niche created by cisplatin *via* ECs would promote M2 macrophage polarization. Therefore, we co-cultured Raw264.7 cells with cisplatin-treated tubular ECs. After co-cultured with cisplatintreated ECs for 48 h, the mRNA level of M1 macrophage marker, iNOS, were significantly dropped while compared to ciaplatin alone or co-culture with ECs alone (Fig. 2A). Furthermore, the mRNA level of M2 markers, argenase-1 and CD206, were elicited significantly in Raw264.7 co-cultured with cisplatin-treated ECs (Fig 2B-C). It demonstrated that cisplatin-induced inflammatory niche *via* ECs could promote M2 macrophage polarization.

### 3.4. M2 but not M1 macrophages have significant effects on promoting cisplatin-induced EMT.

**Figure 3:**
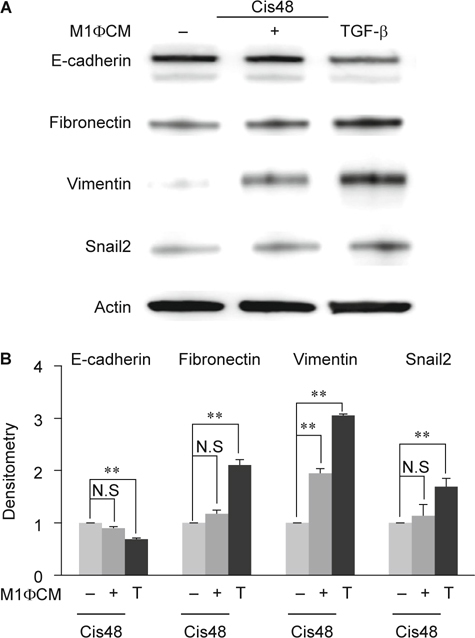

**Figure 4:**
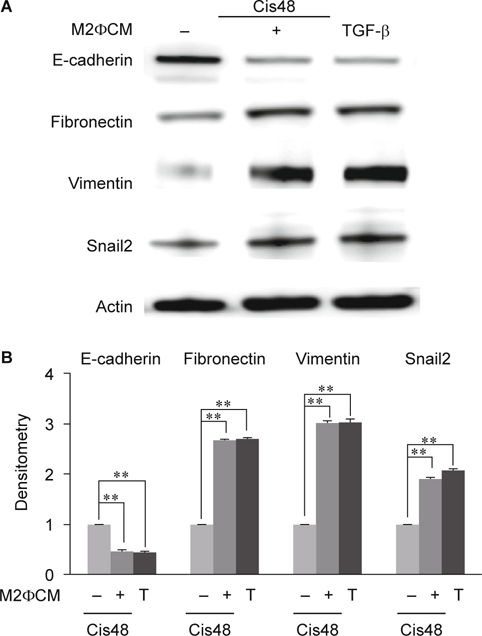

M2 is thought to be pro-fibrotic macrophages due to their secretion of pro-fibrotic cytokine such as TGF-β. Cisplatin-induced inflammatory niche via tubular ECs drive M1 to M2 switch, whether this M1 to M2 switch would turn back to promote the EMT of tubular ECs. We then studied the roles of macrophage polarization on EMT using condition medium of in-vitro induced M1 and M2 macrophages respectively. Compared to cultured in 48h cisplatin alone, tubular ECs cultured with M1 condition medium plus cisplatin had no significant changes in E-cadherin, fibronectin and snail2 excepted vimentin (Fig. 3A-B). TGF-β alone induced more amounts of fibronectin, vimentin and snail2 and suppressed E-cadherin expression significantly than M1 condition medium (Fig. 3A-B). However, tubular ECs cultured with M2 condition medium plus cisplatin significantly enhanced fibronectin, vimentin and snail2 and inhibited E-cadherin while compared to cisplatin alone (Fig. 4A-B). Moreover, compared to M2 condition medium, TGF-β alone could not induce more amounts of fibronectin, vimentin and snail2 and further suppressed E-cadherin (Fig. 4A-B). These indicated that condition medium of M2 macrophages promoted cisplatin-induced EMT in tubular ECs.

## 4. Discussions

In this study, we demonstrated that cisplatin induced incomplete EMT of tubular ECs, but co-culture M2 macrophages, not M1, with cisplatin-treated ECs caused complete EMT. We also founded that co-culture with cisplatin-treated tubular ECs would stimulate fibroblast activation and promote M2 macrophage polarization.

The EMT of tubular epithelial cells was considered as an important source of tubulointerstitial fibrosis. Cisplatin alone or cisplatin plus TNF-α synergetic treatments have been reported to induce ECs EMT or apoptosis [17, 18]. However, Koesters et al have proved that overexpression of TGF-β1, a kind of pro-fibrotic cytokine, in renal tubules in vivo induces tubular autophagy, interstitial proliferation and fibrosis, but not EMT of tubular ECs [31]. These indicated that the cisplatin-induced epithelial EMT should be further confirmed. In present study, we found that cisplatin alone induced incomplete EMT of *in vitro* cultured ECs whereas fibroblasts co-cultured with cisplatin-treated ECs led to fibroblast activation (Fig. 1). It implied that cisplatin-treated ECs played an important role on inflammatory niche. We didn’t further determine the possible cytokines secreted by cisplatin-treated tubular ECs, but it had been reported that TGF-β and IL-4, not IL-6, would be up-regulated by apoptotic human bronchial epithelial cells [32]. This data let us link the cisplatin-treated ECs to M2 macrophage activation. Since M2 macrophage polarization was stimulated by TGF-β and IL-4, our data showed that macrophages co-cultured with cisplatin-treated ECs caused M2 polarization by detecting less iNOS and more Agenase-1 and CD206 expression (Fig. 2).

Macrophages are the only immune cells involved in both inflammatory and recovery phases. Ko and his colleagues had reported that depletion of macrophages by liposome clodronate (LC) during recovery phase attenuated fibrosis in ischemia-reperfusion (IR)-induced renal fibrosis [9]. Recently, macrophages have been considered as two subtypes, pro-inflammation M1 and pro-resolving M2. Some studies demonstrated that M2 type macrophages would reduce inflammation but improve fibrosis in IR injury or adriamycin-induced nephrosis [28, 29, 33]. It had been showed that both M1 and M2 macrophages were depleted by LC but the M2 was preferential after 7 days of IR [29]. Additionally, re-transfer of M2 macrophages following LC treatment would reverse the protective effects of LC on renal fibrosis. All of these results showed that M2 macrophages, not M1, played crucial roles on fibrosis during the recovery phase of IR. However, Wise et al had demonstrated a different role of M2 macrophages. They reported that human mesenchymal stem cells (hMSC) treatment would protect kidney from IR injury and improve renal function by detection of BUN, serum creatinine and kidney injury molecule (kim)-1. But, co-culture of hMSC and M2 macrophages directly or indirectly showed that hMSC promote M2 macrophage development [34]. Therefore, the roles of M2 macrophages on renal fibrosis and functional restoration (regeneration) should be further examined. By detecting several typical marker expressions, our data revealed that cisplatin-treated ECs co-cultured with M2 macrophages, not M1, would present more complete EMT phenomena (Fig. 3 and 4). Taken together, we conclude that cisplatin-treated tubular ECs stimulated M2 macrophage polarization and M2 macrophages would turned back to promote EMT of cisplatin-treated ECs.
